# Transboundary Monitoring of the Wolf Alpine Population over 21 Years and Seven Countries

**DOI:** 10.3390/ani13223551

**Published:** 2023-11-17

**Authors:** Francesca Marucco, Ilka Reinhardt, Elisa Avanzinelli, Fridolin Zimmermann, Ralph Manz, Hubert Potočnik, Rok Černe, Georg Rauer, Theresa Walter, Felix Knauer, Guillaume Chapron, Christophe Duchamp

**Affiliations:** 1 Department of Life Sciences and Systems Biology, University of Turin, Via Accademia Albertina 13, 10123 Turin, Italy; 2 LUPUS—German Institute for Wolf Monitoring and Research, Dorfaue 9, 02979 Spreewitz, Germany; ilka.reinhardt@wolves-germany.de; 3 Department of Biological Sciences, Goethe-University Frankfurt am Main, 60438 Frankfurt, Germany; 4 Centro Grandi Carnivori, Ente di Gestione Aree Protette Alpi Marittime, Piazza Regina Elena 30, Valdieri, 12010 Cuneo, Italy; elisa.avanzinelli@centrograndicarnivori.it; 5 KORA—Carnivore Ecology and Wildlife Management, Talgut Zentrum 5, CH-3063 Ittigen, Switzerland; f.zimmermann@kora.ch (F.Z.); r.manz@kora.ch (R.M.); 6 Department of Ecology and Evolution, University of Lausanne, CH-1015 Lausanne, Switzerland; 7 Biotechnical Faculty, Department of Biology, University of Ljubljana, Jamnikarjeva 101, 1000 Ljubljana, Slovenia; hubert.potocnik@bf.uni-lj.si; 8 Slovenia Forest Service, Večna Pot 2, 1000 Ljubljana, Slovenia; cernerok@gmail.com; 9 Research Institute of Wildlife Ecology, University of Veterinary Medicine Vienna, Savoyenstr. 1, A-1160 Vienna, Austria; georg.rauer@vetmeduni.ac.at; 10 Conservation Medicine Unit, Research Institute of Wildlife Ecology, University of Veterinary Medicine Vienna, Savoyenstr. 1, A-1160 Vienna, Austria; theresa.walter@vetmeduni.ac.at (T.W.); felix.knauer@vetmeduni.ac.at (F.K.); 11 Department of Ecology, Swedish University of Agricultural Sciences, 739-93 Riddarhyttan, Sweden; guillaume.chapron@slu.se; 12 Office Français de la Biodiversité, Department of Research and Expertise, Parc Micropolis, F-05000 Gap, France; christophe.duchamp@ofb.gouv.fr

**Keywords:** population, wolves, Alpine, estimate, monitoring, transboundary

## Abstract

**Simple Summary:**

We conducted a transboundary assessment of the Alpine wolf population over 21 years and across seven countries: Italy, France, Austria, Switzerland, Slovenia, Liechtenstein and Germany. This comprehensive study aimed to elucidate the new population expansion of wolves within the Alpine region after extinction in the area, with an increase in the number of wolf reproductive units (packs and pairs) over 21 years, from the first one in 1993–1994 up to 243 units in 2020–2021. The study highlighted the significance of cross-border cooperation in understanding the recolonization process of the wolf Alpine population. This transboundary evaluation not only contributes to scientific knowledge but also offers a foundation for informed decision making to favor the coexistence of wolves and human communities across the anthropized landscapes of the Alps.

**Abstract:**

Wolves have large spatial requirements and their expansion in Europe is occurring over national boundaries, hence the need to develop monitoring programs at the population level. Wolves in the Alps are defined as a functional population and management unit. The range of this wolf Alpine population now covers seven countries: Italy, France, Austria, Switzerland, Slovenia, Liechtenstein and Germany, making the development of a joint and coordinated monitoring program particularly challenging. In the framework of the Wolf Alpine Group (WAG), researchers developed uniform criteria for the assessment and interpretation of field data collected in the frame of different national monitoring programs. This standardization allowed for data comparability across borders and the joint evaluation of distribution and consistency at the population level. We documented the increase in the number of wolf reproductive units (packs and pairs) over 21 years, from 1 in 1993–1994 up to 243 units in 2020–2021, and examined the pattern of expansion over the Alps. This long-term and large-scale approach is a successful example of transboundary monitoring of a large carnivore population that, despite administrative fragmentation, provides robust indexes of population size and distribution that are of relevance for wolf conservation and management at the transnational Alpine scale.

## 1. Introduction

The 21st century is a period in which the recovery of large carnivores has been widely documented in Europe [1]. Wolves (*Canis lupus*), after being extensively reduced in the 1800–1900s (Ripple et al. 2014), have been naturally recolonizing the territories of several European countries [2,3]. Considering the highly controversial status of the species, wildlife managers and governments often face a hostile political landscape to manage the species at the country level [4,5], usually based on data from national monitoring programs [6,7,8]. However, wolf expansion in Europe is occurring over administrative boundaries and the majority of the wolf populations are shared between countries [1]. In this framework, Linnell et al. (2008) [9] developed the “Guidelines for Population Level Management Plans for Large Carnivores in Europe”, endorsed by the European Commission, where the need to develop monitoring programs at the population level is highlighted for wolves in particular. Wolves are known to roam over large territories of up to several hundred square kilometers [10], which may extend beyond administrative borders [11], and dispersers are able to move over hundreds of kilometers [12,13,14,15]. Therefore, for proper conservation and management issues, it is useful to consider the whole population as a management unit rather than population segments occurring within national boundaries [9]. The first step to actually manage wolves at the population level is using a monitoring program at such a scale. Even though attempts to officially introduce this concept date back to 2008, few examples of population-level monitoring programs have been developed so far. The greatest success has been achieved in Scandinavia, where large carnivores’ populations have been estimated using cross-national genetic monitoring [16]; in this case, only two countries were involved (i.e., Sweden and Norway). Other attempts have been conducted for the central European wolf population between Germany and Poland [17] and for the Dinaric population between Slovenia and Croatia. However, these approaches were not fully implemented, considering that monitoring standards were agreed upon [17] but not yet used and implemented for joint evaluations of the population.

Although genetically and demographically connected to the Italian wolf population in the Apennines [18], and to the Dinaric–Balkan population in the East [19], wolves in the Alps are considered as a distinct management unit (Alpine population) because their ecological and socio-economic contexts strongly differ from those of the Apennine population [9]. Wolves began naturally recolonizing the south-western Alps at the beginning of the 1990s from the north Apennines wolf subpopulation after being extirpated from the Alps in the early 1900s. Simulations of the wolf natural recolonization process showed that a total of 8–16 effective founders explained the genetic diversity observed in the western Alps in the first years of recolonization [18]. Over the years, dispersals from the Apennines have been documented towards the north-western Alps and central Alps through different countries [14,18]. A similar process of recolonization began in the eastern Alps as individuals from the Dinaric–Balkan population dispersed and reached the Alps as well [19]. This expansion is demonstrated by several dispersers that were genetically monitored across countries [14]. The case of a GPS-collared male wolf from a Slovenian pack that traveled through Austria to finally settle with a wolf female in the Italian eastern Alps [13] also gives a good example of the species’ capacity. Hence, the recolonization of wolves in the Alps interests the entire mountain chain of seven countries: Italy, France, Austria, Switzerland, Slovenia, Liechtenstein and Germany, making the development of a unique and coordinated monitoring program particularly challenging and exceptional worldwide.

Here, we describe how we developed standardized monitoring among research groups and implemented an integrated approach among the seven countries, allowing us to closely monitor the recolonization process of the transboundary Alpine wolf population over 21 years. We first founded a technical working group, the Wolf Alpine Group (WAG), to ensure strong coordination among wildlife experts of the different countries. Under the umbrella of the WAG, researchers developed uniform criteria for the assessment and interpretation of field data collected in the frame of different national monitoring programs. This standardization allowed for data comparability across borders and the joint evaluation of distribution and trends of abundance at the population level [20]. Based on these joint monitoring standards, we produced maps of area of occurrence and distribution, estimated population size indices and growth rate, thus providing robust data with direct relevance for wolf conservation and management at the transboundary Alpine scale. We examined the patterns of expansion in the wolf recolonizing population in the Alps over 21 years. This long-term and large-scale approach is a successful example of conservation practice in the Alpine region, where despite administrative fragmentation, a wide roaming and transboundary population of a large carnivore is monitored with a coordinated strategy.

## 2. Materials and Methods

**Figure 1:**
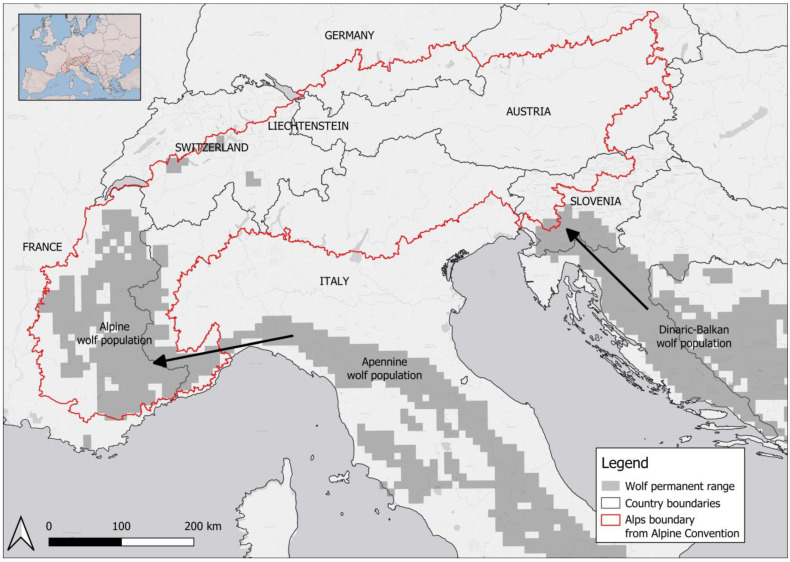
Study area defined by the Alps range as reported by the Alpine Convention (red line), country boundaries and indications on the Apennine and Dinaric–Balkan wolf populations that originated the Alpine wolf population based on Chapron et al. 2014.

The Wolf Alpine Group (WAG). In 2001, a first workshop was organized in France gathering experts from France, Italy and Switzerland concerned with the study of the recent recolonization of the wolf population over the Alpine area, defined by the Alpine Convention (Figure 1). The main objective was to establish an effective collaboration and share experiences among the three countries in order to exchange the first scientific data. The WAG then started to effectively monitor the size of the transboundary wolf population in the Alps as a whole. Since then, a strong collaboration among scientists has been set up and significant progress has been made over time, especially regarding information exchanges and shared methodologies. Twenty-one years after the first workshop, the Wolf Alpine Group met for the 12th time in 2022, with the main goal of updating the wolf population assessment in the Alps across the different countries. The WAG extended over the years following the wolf recolonization process. The group has gathered researchers since 2010 from Austria, Germany and Slovenia, and since 2022 from Liechtenstein. Therefore, the WAG is currently represented by experts from each of the seven countries of the Alpine range. Associate research groups, such as the laboratories in charge of genetic analysis in each country, also regularly contribute to the WAG outputs [20,21]. Details on the WAG meetings, technical reports and process evolution over time are described in Supplementary Material S1 (SM1).

Agreed standard criteria for classifying and interpreting wolf presence data. Within the WAG, researchers from each country aimed to harmonize the data evaluation and interpretation in order to estimate the area of occurrence and population size across borders. We defined standard criteria for classifying (Table 1) and interpreting data collected in the field, setting common sampling unit definitions (Table 2). We adopted the shared standard criteria for classifying wolf observations based on 3 categories: hard evidence (C1), confirmed observations (C2) and unconfirmed observations (C3) [22] (Table 1). All observations were checked for their genuineness (i.e., the possibility of intentional deception must be ruled out). Signs of presence that could not be evaluated because of lack of minimum technical descriptions were discarded.

**Figure 2:**
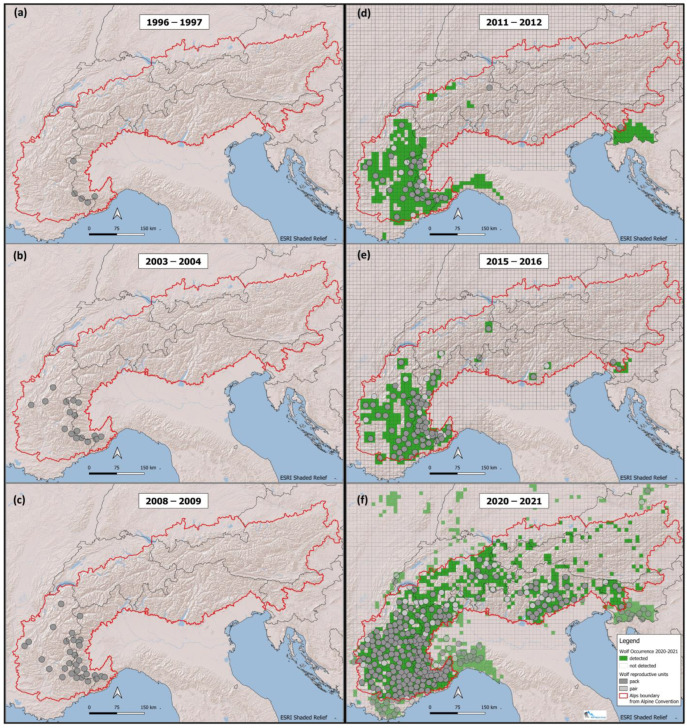
Distribution of reproductive units in the Alps in 1996–1997 (**a**), 2003–2004 (**b**) and 2008–2009 (**c**), together with wolf occurrence for 2011–2012 (**d**), 2015–2016 (**e**) and 2020–2021 (**f**).

The WAG agreed that the basic reproductive units of a wolf population are packs and pairs [10]; considering that up to 80–85% of the wolves of a population belong to packs [10], we used packs as the main sampling unit to monitor population size and trends over time. As we did not aim to estimate the total number of individuals but the number of reproductive units, dispersers or solitary individuals were not reported in the evaluation of the population trend. However, their presence, if detected, was included in the area of wolf occurrence. Packs or pairs were defined as “transboundary” (TR) when their presence on both sides of the border was documented with genetic matches, or “likely Transboundary” (LTR) based on the interpretation of the spatio-temporal distribution of wolf signs; in both cases, they are represented by a circle crossed by a national boundary in Figure 2. Additional details are given in Supplementary Material S2 (SM2).

Wolf population evaluation and trend. We estimated area of occurrence and population size over time by evaluating the area of documented occurrence and the number of packs/pairs detected. Wolf occurrence is the area occupied by the species. It is described as the grid cell number and distribution of the European Environment Agency (EEA) 10 × 10 km reference grid (LAEA89 datum, https://www.eea.europa.eu/en/datahub/datahubitem-view/3c362237-daa4-45e2-8c16-aaadfb1a003b (accessed on 12 November 2023)), where the presence of the species was confirmed by at least 1 C1 observation or 2 C2 observations within the monitoring year (from 1st of May to the 30th of April; Table 2). The EEA reference grid is a pan-European grid to be used for reporting species distribution under Article 17 of the Habitats Directive (Council Directive 92/43/EEC).

Although the grid of wolf occurrence can be reported beyond the border of the Alpine convention, as for the last sampling year 2020–2021, the trend of the occupied area was calculated within the Alpine range only to focus on the transboundary Alpine population entity [9]. We considered changes in the number of wolf packs and pairs as a biologically meaningful index of population trends, such as in other wolf monitoring systems worldwide [2,23,24]. However, such estimates were not available every year for every country due to funding constraints. Total estimates were continuously available from 1993–1994 to 2011–2012 when the wolf population was smaller, then only for given years such as 2015–2016 and 2020–2021.

We estimated population growth rate using a Bayesian hierarchical state-space model. Our model treated process and observation separately. In the process model, we assumed that the wolf population grows exponentially with rate λ and with a stochastic process error added on the deterministic prediction of the model. The process model was linked to the observation model in which the number of reproductive units was drawn from a Poisson distribution whose parameter was gamma-distributed with a mean equal to the prediction of the process model with an observation error term. We fitted the Bayesian model to the time series of wolf reproductive units in the Alps, choosing uninformative priors and running 8 Monte Carlo Markov chains (100,000 iterations, thinning by 10 after adapting and updating for 10,000 iterations) with JAGS [25]. Maps were developed in QGIS version 3.24 [26], and all analyses were performed in R software version 4.1.3 [27].

## 3. Results

### 3.1. Wolf Occurrence and Distribution of Packs/Pairs

Based on the agreed standard criteria (Table 1 and Table 2), we documented the transboundary distribution of reproductive wolf units in the Alps (Figure 2) for 1996–1997 (a), 2003–2004 (b) and 2008–2009 (c), and wolf occurrence for 2011–2012 (d), 2015–2016 (e) and 2020–2021 (f). The transboundary dataset on wolf occurrence is reported for 2020–2021 over the entire range, and also beyond the Alpine area (Figure 2). The first five wolf packs in 1996–1997 were documented in the south-western part of the Alps, two of which were transboundary territories (Figure 2a). Up to 2009, subsequent territories were established in the surroundings, forming the source recolonization area for the Alps (Figure 2b,c). From 2011 to 2016, the first territories of wolf reproductive units were formed outside the western recolonization area, in the central and south-eastern Alps. This reproductive nucleus, also stemming from the Dinaric–Balkan population, represented a new source for the recolonization of the Alps, accelerating the process especially in the south-eastern and central Alps (Figure 2d,e). In addition to dispersing individuals from the fast-growing population segment in the western Alps, more wolves from the south-eastern mountain range now dispersed to the north and the west as indicated by the increase in the area of occurrence in Figure 2e,f. In this process, the first reproductive units were found in the central Alps of Switzerland in 2011–2012 (Figure 2d), and of Italy in 2014–2015 (Figure 2e). Some of these initial recolonizing events in the central Alps, far away from the source western Alps area, served as stepping stones which allowed subsequent colonization in the surrounding areas (Figure 2f). In 2020–2021 wolf occurrence concerns all of the 7 Alpine countries, indicating a strong trend of potential recolonization over the entire Alpine chain. The western part of the Alps has been almost entirely occupied and has the highest pack density in the Alps, particularly in the south-western Italian part, and north and westward in France (Figure 2f). In these areas, wolves have reached hills and expanded beyond the Alpine mountain range. In the same time period, the Dinaric population has also expanded northward. Figure 2 shows the international dimension of the wolf Alpine population and the transboundary reproductive units detected. The area of occurrence within the Alpine range increased from 41,100 km^2^ in 2011–2012 (Figure 2d) to 71,500 km^2^ in 2015–2016 (Figure 2e) and up to 91,000 km^2^ in 2020–2021 (Figure 2f).

### 3.2. Wolf Population Trends from 1993–1994 to 2020–2021

**Figure 3:**
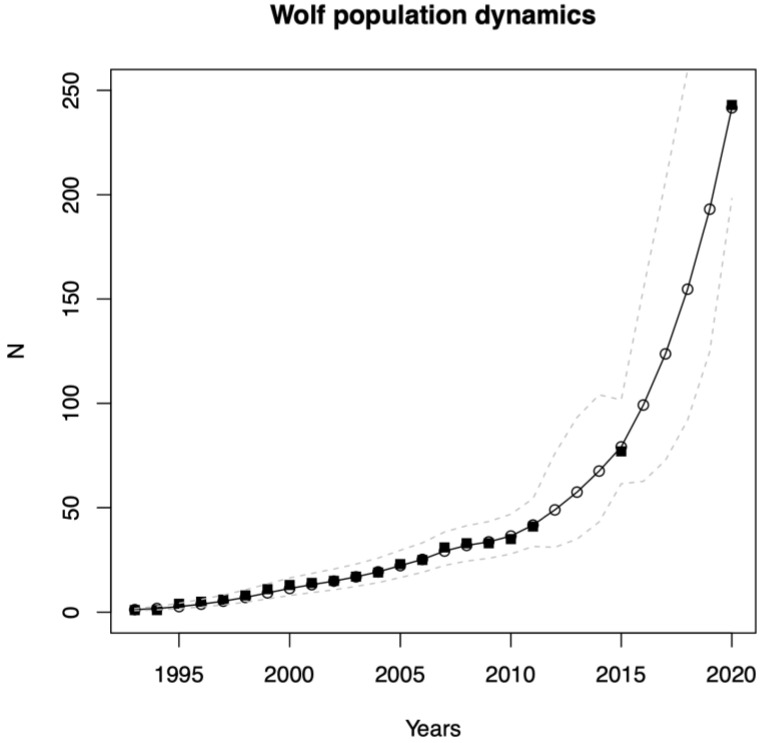
Hierarchical Bayesian exponential model fitted to the time series of wolf reproductive units *N* (black squares are collected data from 1993–1994 to 2020–2021, white circles and continuous black line are medians of the model-inferred population sizes when direct estimates were not available and dashed lines are 95% credible intervals).

We documented the increase in the number of wolf reproductive units (i.e., packs and pairs) occurring in the Alpine area, from 1 unit in 1993–1994 to 243 units in 2020–2021. Using the exponential growth population model fitted to the time series of reproductive units, we estimated the annual growth rate λ = 1.22 ± 0.05 (Figure 3). Distinguishing between adjacent packs and pairs is a prerequisite for a minimum count of reproductive units and hence for estimating a robust index of population size. However, in high-density areas, this requires lots of effort and genetic information. Therefore, this exercise could not be conducted regularly by every country every year, but was concentrated in given years. Due to limited funding, this was the case for several years in Italy and Slovenia. Hence, the number of wolf packs and pairs was not continuously quantified at the Alpine scale (Figure 3).

In 2015–2016, we recorded 65 wolf packs and 12 pairs over the Alps, with the great majority of them located in the western part between Italy and France (Figure 2e and Figure 3). In Italy, we documented 27 packs and 8 pairs; in France, 31 packs and 3 pairs; in Switzerland, 1 pack and 1 pair; and in Slovenia, 2 packs. Moreover, we documented one transboundary pack between Switzerland and Italy and three between Italy and France. Pairs represented 15,6% of the reproductive units detected.

In 2020–2021, we documented a total of 206 packs and 37 new pairs for a total of 243 reproductive units in the Alps (Figure 3). Pairs represented 15.2% of the reproductive units detected, about the same proportion compared to the previous period. The major contributions to the increase over the years were given by France (52%) and Italy (40%), which are also the countries where the recolonization in the Alps started the longest time ago. In 2020–2021, considering only the Alpine area, in Italy we documented 82 packs and 16 pairs; in France, 107 packs and 11 pairs; in Switzerland, 8 packs and 8 pairs; and in Slovenia, 5 packs. Moreover, we documented two (one pack and one pair) transboundary reproductive units between Switzerland and Italy, two between Italy and France, one pack between France and Switzerland, and one pack between Italy and Slovenia (Figure 2f).

## 4. Discussion

Transboundary approaches are becoming an emerging priority for biodiversity conservation [28]. Many transboundary landscapes are species-rich because international borders often overlap with mountainous terrain or other complex landscapes, which naturally support high richness and endemism [29]. The distribution of large carnivores in Europe covers many transboundary landscapes [1], with differences in policy and management strategies among neighboring countries [30]. The monitoring of the recolonization process of the wolf in the Alps interests seven countries: Italy, France, Austria, Switzerland, Slovenia, Liechtenstein and Germany, making the overall coordination particularly challenging. Starting from each national wolf monitoring system and strategy [21,31], we identified a common baseline that allowed us to monitor wolves in the Alpine area as a unique biological entity.

Since the ecology of the species is based on the social and territorial dynamics of packs and pairs [10], this metric is particularly meaningful to document the persistence and expansion of a wolf population [32]. Therefore, the number of reproductive units is especially relevant for wolf population monitoring and gives a robust index of population trends [33]. By focusing on the reproductive units as the main population index, the WAG follows the example of other wolf monitoring systems worldwide, such as in North America in the Great Lakes region [34], the Northern Rocky Mountains [24] and Idaho [35], and in Europe in Germany [2] and Sweden [33]. Hence, the key element of the standardization was to agree on the definitions of reproductive units and on the data needed to confirm their presence. Although the same methods are used among the different countries (e.g., non-invasive genetic methods, wolf howling, collection of signs of presence), the way they are implemented often differs because of the effort required given the size of the population, or because of the different countries’ priorities according to the population status. We therefore focused on the common interpretation of the recorded data. This joint data interpretation allowed us to combine the results of the individual country monitoring programs across borders. Special attention was paid to potential cross-border territories to avoid double counts of packs.

The combined transboundary Alpine dataset showed a substantial increase in the number of packs and pairs, especially in recent years, following an exponential growth. This pattern has also been observed in other wolf recolonization processes after extinction, both in the Central European population in Germany and Poland [2,36], and in North America in the Great Lakes region [34] and in Northern Rocky Mountains [24,35]. In the Yellowstone area, after a similar initial increase, the population stabilized [37]. The main contribution and increase in population occurred in the western Alps of Italy and France, where wolf pack density is highest. The western part of the Alps is almost completely occupied, and represents the source of wolf recolonization of the entire Alps. At the same time, initial recolonizing packs in the central Alps, far away from the western Alps source area, served as stepping stones allowing subsequent colonization of the surrounding areas. The observed population growth was accompanied by a significant spatial expansion of the population, recognizable by the increase in the area of occurrence represented by the number of 10 × 10 km grid cells where wolf presence was confirmed. Similar to the population trends, we could also follow the spatial spreading of the population over the entire Alpine chain because we shared the same definitions and criteria for confirming the presence of the species across national borders. Most of the presence signs found were left by wolves living in packs or pairs; however, at the forefront of the recolonization process, part of the grid cells occupied were attributable to dispersing or single resident individuals. Those individuals were not included in the population size index (as they do not contribute to reproduction); their presence is accounted for in the area of occurrence maps reflecting the spatial population dynamic.

We demonstrated that the number of reproductive units and the area occupied by wolves can be collected and combined across different countries in a consistent way. Instead of combining and discussing the entire raw datasets, countries within the WAG could just merge the results of their national monitoring programs in the certainty that these results are comparable across national borders on the basis of the agreed standard criteria and definitions. This approach is optimal and efficient for long-term monitoring of large-scale populations divided within several countries, such as for wolves in the Alps. This approach gives biologically meaningful estimates of the population status as is required, for instance, by the EU Habitats Directive reporting.

When an estimate of population size given by the number of individuals is required, a cross-border approach is more challenging and will likely require combining raw datasets and measures of effort. At country levels, or smaller scales, large carnivore monitoring programs use non-invasive genotyping for estimations of population size from capture–recapture models [38,39], or more recently, spatial capture–recapture (SCR) approaches [40,41,42], to obtain accurate estimates of population size with measures of precisions using Confidence Intervals or Credible Intervals. These approaches have also been used at the country level in France [39] and in Italy [7]. However, it would not be appropriate to combine these resulting estimates for a transboundary population evaluation which shares individuals without combining raw datasets because it would overestimate the population by double-counting animals [43]. To obtain a single, accurate estimate of population size on a transboundary scale with CR or SCR models, it would be necessary to merge genetic datasets and have a robust and comparable measure of the effort for the different sampling areas [7,16]. However, combining entire datasets and genotypes analyzed from several genetic laboratories would be much more expensive and time-consuming, and common genetic monitoring standards (same method and markers) should be developed [44]. This work could be prone to errors and lead to biased results [45].

Another approach to estimating the individual number of wolves is to develop a conversion factor that translates the number of reproductive units into the number of wolves [46]. However, to develop a robust conversion factor, detailed data from the investigation area are necessary [46]. If these conversion factors were applied over several countries at different population stages (saturated versus expansion front), the resulting CI would be high. Compared to the aforementioned methods for estimating wolf population size, the indexes provided in our study are more comparable, additive and efficient. At this advanced stage of the wolf’s transboundary expansion over the Alps and in order to document trends, we believe it is more practical to track indices of wolf presence and reproductive units in space and time (i.e., grid of occurrence, number of packs/pairs) instead of trying to estimate wolf population size as individual numbers, which would be much more demanding in terms of funds and effort.

In perspective, with further expansion of the wolf population, new challenges are expected. It is becoming more difficult to distinguish adjacent packs in high-density areas. Adjacent packs can be distinguished only with genetic identification (i.e., pack pedigree) or through simultaneous evidence of pack reproduction. This requires intensive application of genetic analysis, or simultaneous camera trapping and wolf howling with either active or passive acoustic monitoring [47,48]. This will require a substantial increase in field effort and funds, and cannot be conducted regularly by every country. Hence, the number and distribution of reproductive units as well as the area of occurrence will not be documented at the Alpine scale on a yearly basis. As a consequence, it will be fundamental to synchronize the country’s evaluations within the WAG as has already been the case in recent years. We also recommend common genetic monitoring standards and techniques among the different country’s laboratories in order to facilitate genotype comparison.

The wolf population in the Alps is expanding in every country beyond the Alpine range, towards the mountains, along rivers and down to the plains [7]. Each country will continue to monitor wolves at the country level within and beyond the Alpine area. Wolf populations in Europe are expanding and re-connecting [49]; hence, a comparable Europe-wide approach to determine the status of the different wolf management units will soon be reasonable and necessary. Our cross-border monitoring approach could then be extended to a large-scale metapopulation evaluation.

## 5. Conclusions

We highlighted the exceptional long-term strict collaboration developed among researchers of seven countries in the Alps, which allowed for the development of a unique transboundary approach useful to combine large scale datasets. This experience is key to shaping common strategies and unifying datasets of other transborder populations in Europe and worldwide with minimum effort. Key recommendations for large-scale transboundary wildlife population evaluations include the need for developing species-specific monitoring standards with detailed data interpretation criteria to allow population assessments without the need for merging raw datasets. This approach could be scaled up to even larger scales, metapopulation evaluations, and adopted to other continents and species. We created a large-scale network of researchers that is now available for long-term biodiversity conservation to overcome the high administrative fragmentation in practices by various government offices. The infrastructure put in place will guarantee future population assessments and also put new challenges into perspective. The creation of the WAG has also been key for raising awareness of the international aspects of the wolf population, favoring exchanges of best practices of a very controversial species, and providing an important asset for long-term biodiversity conservation.

## Figures and Tables

**Table 1:** Classification criteria of wolf signs of presence reported for the wolf monitoring. The letter “C” stands for “category”. The numbers 1, 2 and 3 denote the level of validation for an observation as hard evidence (C1), confirmed observation (C2) and unconfirmed observation (C3).

C1	C2	C3
Animals captured or rescued alive	Documented tracks with typical trend/pattern followed for at least 100 m	Tracks followed for less than 100 m in snow or single footprint
Dead animals	Documented scats checked by an expert, or found on a track classified as a C2 observation	Scats not confirmed by an expert and not associated with snow tracks
Whatever DNA evidence confirms the biological sample (i.e., scats, hair, blood, urine, saliva, regurgitate, bones)	Predation signs with typical bites and/or consumption, only if combined with other C2 data	Heavily eaten kills, livestock depredations not technically documented, or not combined with other C2 data
Indirectly certified presence signs from DNA evidence (e.g., tracks on snow)	Howl with wolf pups’ presence, checked by an expert	Single howls
Good-quality video and photos		Sightings not supported by photos or videos
Telemetry locations		Bad-quality videos or pictures which do not allow secure identification of species

**Table 2:** Common definitions used in wolf population evaluation and agreed criteria for data interpretation at the Alpine level.

Unit	Definition	Data Needed
Monitoring year	Biological year for wolves (from reproduction to next reproduction)	From the 1st of May to the 30th of April
Pair	Only 1 male +1 female holding a territory and traveling together but not (yet) having reproduced	C1 that confirms the pair bonding together: video/photo/genetic, or a track of the pair with genetic proof of the couple
Pack	Reproductive unit identified by either pup occurrences or by at least 3 individuals traveling together and holding a territory	Reproduction confirmed with one C1 or C2, or at least 2 independent C2s showing the pack traveling together (tracks), or ≥3 individuals confirmed by C1 (genetics/photo/video)
Wolf Occurrence (Cell)	10 × 10 km cell (EEA grid) where the species has been detected within the monitoring year	At least one C1 or two independent C2s
Representation of the Territory	Area held by the resident wolf/wolves to show its approximate localization over space	Circle of about 200 km^2^ centered on the centroid of the Minimum Convex Polygon (MCP) constructed on the collected C1–C2 wolf signs
Adjacent packs	Two adjacent packs need to be clearly distinguished to be considered two units (or more) by either: ○ Genetic data for pack identification; ○ Simultaneous proof of reproduction; ○ Telemetry data from radio collared wolves belonging to one of the adjacent packs.	C1 data from genotyping showing pedigrees and space separation between members of the packs; or C2 data from simultaneous rendezvous site detections in each of the territories
Mature individual	Wolf of ≥22 months, considered adult	C1 data
Pup	Wolf in its first year of life. The transition from pup to yearling is considered on 1st May.	C1 data
Yearling	Wolf in its second year of life	C1 data

## Data Availability

Data are contained within the article and Supplementary Materials.

## Supplementary Materials

The following supporting information can be downloaded at: https://www.mdpi.com/article/10.3390/ani13223551/s1, Supplementary Material S1 (SM1). The Wolf Alpine Group (WAG), Table S1. List of the Wolf Alpine Group (WAG) meetings/workshops and the relative location, number of people present, and countries represented at each meeting. Supplementary Material S2 (SM2). Detailed description of the methods adopted by the Wolf Alpine Group for the monitoring of the wolf alpine population. References [10,20,21,22,50,51,52,53,54,55,56,57] are cited in the Supplementary Materials.
