# Supplementary material for: Transboundary Monitoring of the Wolf Alpine Population over 21 Years and Seven Countries
Source: Animals (Basel). 2023 Nov 17;13(22):3551. doi: 10.3390/ani13223551 (PMC10668717; doi:10.3390/ani13223551)
Supplement: Supplementary file 1 [file animals-13-03551-s001.zip › animals-2643384-supplementary.pdf]

SUPPLEMENTARY Material

SM1. The Wolf Alpine Group (WAG)

Facing transboundary wolf recovery from Italy to France since the early 90's, the needs of exchanging data and experiences was significant. The Wolf Alpine Group was created in 2001 first to exchange field cases and data and to start monitoring the species on a common basis between the first 3 main countries of concern (France, Italy and Switzerland at this stage). Then the group was constructed as an expert group meeting every 2 years to exchange experiences and conclude on a common way of doing without any formal or juridical consistency (see table of meetings). Then the group has grown as the wolf population in the Alps expanded, with Slovenia and Austria joining the group in 2013. From that point the WAG became the reference group of experts following the recommendations of the Large Carnivore Initiative for Europe (LCIE) who defined the "alpine population" as a functional population unit. The group answered the requisites of the European Council for the population status as well as the LCIE international map updates. Published technical reports have been the products of the workshops (WAG 2008, 2014, 2018, 2022, 2023). All technical reports have been co-signed on behalf of the Wolf Alpine Group.
The wolf alpine group developed standards for baseline monitoring as well as promoting genetic standards and improvement between academic labs (WAG 2022). Thanks to various financial support such as LIFE projects, major insights have been produced over the 12 workshops (Table S1).

Table S1.List of the Wolf Alpine Group (WAG) meetings/workshops and the relative location, number of people present, and countries represented at each meeting.

Year of WAG Workshop	Location	Minimum number of experts present	Countries represented
2001: 1st meeting	Briancon (France)	8	France, Italy, Switzerland
2003: 2nd meeting	Boudevilliers (Swiss)	18	France, Italy, Switzerland
2004: 3rd meeting	Entracque (Italy)	12	France, Italy, Switzerland
2005: 4th meeting	St Martin (France)	13	France, Italy, Switzerland
2007: 5th meeting	La Fouly (Swiss)	20	France, Italy, Switzerland
2010: 6th meeting	Entracque (Italy)	14	France, Italy, Switzerland
2013: 7th meeting	Jausiers (France)	16	France, Italy, Switzerland, Slovenia, Austria
2015: 8th meeting	Bormio (Italy)	29	France, Italy, Switzerland, Slovenia, Austria, Germany
2018: 9th meeting	Podcerkev (Slovenia)	12	France, Italy, Switzerland, Slovenia, Austria, Germany
2020: 10th meeting	On line	24	France, Italy, Switzerland, Slovenia, Austria, Germany
2022: 11th meeting	On line	12	France, Italy, Switzerland, Slovenia, Austria, Germany
2023: 12th meeting	Barcelonnette (France)	10	France, Italy, Switzerland, Slovenia, Austria, Germany

Technical reports produced by the Wolf Alpine Group:

Wolf Alpine Group (2008). Wolves in the western Alps: Monitoring and Conservation Status. First report to the Permanent Committee Available at http://www1.nina.no/lcie_new/pdf/635422305235918773_First_report_to_the_Permanent_Committee_-_english.pdf

Wolf Alpine group (2014). Wolf population status in the Alps: pack distribution and trend up to 2012. 6p. Available at http://www.lcie.org . 2014 June.

Wolf Alpine Group (2018) Wolf population status in the Alps: pack distribution and trend up to 2016, with focus on year 2015-2016. Available at http://www.lcie.org . 2018 March.

Wolf Alpine Group (2022). The integrated monitoring of the wolf alpine population over 6 countries. Report for LIFE WolfAlps EU project LIFE18 NAT/IT/000972, Action A5. Available at https://www.lifewolfalps.eu/wp-content/uploads/2022/05/A5_-LWA_WAG_Monitoring-Standards-of-the-Wolf-alpine-population.pdf

Wolf Alpine Group (2023). The wolf Alpine population in 2020-2022 over 7 countries. Technical report for LIFE WolfAlps EU project LIFE18 NAT/IT/000972, Action C4. Available at https://www.lifewolfalps.eu/wp-content/uploads/2023/05/C4_WAG_Deliverable_C4_2020_2022.pdf

SM2. Detailed description of the methods adopted by the Wolf Alpine Group for the monitoring of the wolf alpine population

Standard criteria for classifying wolf observations

The SCALP (Status and Conservation of the Alpine Lynx Population) criteria were first published in 2003 (Molinari-Jobin et al. 2003) and are now used worldwide for different species (e.g. Reinhardt et al. 2015, Zimmermann 2019, Ghoddousi et al. 2022). SCALP is a conservation initiative that developed standardised criteria for the interpretation of data for alpine lynx monitoring. WAG researchers adopted standard criteria for classifying wolf observations, based on the SCALP ones. Under SCALP, observations are classified according to their verifiability into:
- C1 = hard facts,
- C2 = confirmed observation,
- C3 = unconfirmed observations.

These criteria have been adapted to wolves and other species with adjustments and adopted by different countries in Europe (Kaczensky et al. 2009, , Reinhardt et al. 2015, Marucco et al. 2020). Hereafter, we defined the agreed SCALP criteria adapted to wolves for the monitoring of wolves in the Alps (WAG, 2022).

A few preconditions apply:
● at least one experienced person must be available for the evaluation of field data, .
● an operator is "experienced" if extensive experience in the collection of field data on wolves has been acquired, meaning that there is the ability of recognising and interpreting signs left by wolves in the field. The operator must prove to have recently taken part in recognised scientific wolf surveys.
● all observations must be verified to rule out the possibility of intentional deception. An example of this last case is given by pictures taken with the cell phone that are attributed to false locations. For this reason, every picture needs to be checked by an expert, not only to recognize the species, but also to verify the precise location by field verification, and the observer by direct interview.

The letter "C" indicates "category". The numbers 1, 2 and 3 identify the level of validation for an observation.

- C1: Hard evidence, i.e., evidence that confirms the presence of a wolf, like dead animals, genetic proof, animals captured or rescued alive, telemetric or telemetry locations, good quality video/photo.
- C2: Confirmed observation, i.e.indirect signs confirmed by an experienced observer as being produced by the wolf, like scats, kills and howls with wolf pups, or tracks followed for at least 100 m, scats, . The experienced operator can either directly confirm the signs in the field or do it base on documentation (the details on the needed documentation is country-dependent).
- C3: Unconfirmed observation, i.e. observations that are not confirmed by an experienced person or which by their nature cannot be confirmed. Examples include all signs that are not clear, not recognisable, or incompletely documented. For example, signs that do not provide a clear picture (e.g. a only a single footprint, heavily eaten kills); and signs that cannot be verified (for example sightings which are not supported by photos).

False observations are not considered and are entirely ruled out. An example of this last case is given earlier, by pictures taken with the cell phone that are attributed to false locations, which need to be regularly verified by an expert, and if the case excluded from the database.
Table 1 from the main text summarizes the classification of wolf signs in each C category.

Definitions and criteria for data interpretation

The WAG defined criteria for data interpretation to combine results at the alpine level and agreed on specific definitions and parameters used in wolf monitoring over the Alps, updating definitions used in 2018 (WAG 2018, 2022). The important reproductive social units of a wolf population are packs and pairs (Mech and Boitani 2003). A pack is defined by at least ≥ 3 individuals travelling together while holding a territory or by pup occurrence. A pair is defined as one male and one female who mark their territory. Dispersers or solitary individuals are not considered in the evaluation of the population trend as represented by the number of reproductive units, but are included in wolf occurrences. Packs have been defined as “transboundary” (Tr) once hard evidence is documented with genetic matches in areas across national borders, or as “likely transboundary” (LTr) without hard facts but based on the interpretation of the spatial distribution of wolf signs.

We acknowledge that It is difficult to distinguish adjacent packs when an area becomes saturated with wolf packs. Hence, criteria for distinguishing adjacent packs have been defined at the international scale after Reinhardt et al. (2015). Simultaneous camera trapping, or intensive application of genetic analysis, or simultaneous wolf howling are necessary to distinguish one pack from an adjacent one in high pack density. Adjacent packs can be distinguished only with the genetic identification of each pack by constructing pack pedigrees, or through simultaneous evidence of pack reproduction in both areas (through videos/photo or pups’ howls or telemetry).
In Table 2 from the main manuscript the common definitions used in wolf population evaluation and agreed criteria for data interpretation at alpine level are presented.

Literature cited

Ghoddousi, A., Abolghasemi, H., Arianejad, M., Mozafari, G.G., Joolaee, L., Mousavi, M., Nayeri D., Chahartaghi N.R. , Raza, H., and P. Sepahvand. (2022). Distribution and status of the Persian leopard in its western range. CATnews Special Issue 15 Summer 2022.

Kaczensky, P., Kluth, G., Knauer, F., Rauer, G., Reinhardt, I., Wotschikowski, U. (2009). Monitoring of Large Carnivores in Germany. BfN-Skripten 251. 99pp.

Marucco F., La Morgia V., Aragno P., Salvatori V., Caniglia R., Fabbri E., Mucci N. e P. Genovesi., (2020). Linee guida e protocolli per il monitoraggio nazionale del lupo in Italia. Realizzate nell'ambito della convenzione ISPRA-Ministero dell'Ambiente e della Tutela del Territorio e del Mare per "Attività di monitoraggio nazionale nell'ambito del Piano di Azione del lupo".

Mech L.D. and Boitani L. (2003) Wolves: Behavior, Ecology, and Conservation. Chicago & London: University of Chicago Press.

Molinari-Jobin, A., Molinari, P., Breitenmoser-Würsten, C., Wölfl, M. et al. (2003). Pan-Alpine Conservation Strategy for the Lynx. Nature and environment No. 130. Council of Europe Publishing, Strasbourg

Reinhardt, I., Kaczensky, P., Knauer, F., Rauer, G. et al. (2015). Monitoring von Wolf, Luchs und Bär in Deutschland. 2., überarbeitete Version. BfN-Skripten.

Wolf Alpine Group (2018) Wolf population status in the Alps: pack distribution and trend up to 2016, with focus on year 2015-2016. Available at http://www.lcie.org . 2018 March.

Wolf Alpine Group (2022). The integrated monitoring of the wolf alpine population over 6 countries. 2022, Report for LIFE WolfAlps EU project LIFE18 NAT/IT/000972, Action A5.

Zimmermann F. (2019). Monitoring von grossen Beutegreifern.In Wolf, Luchs und Bär in der Kulturlandschaft.Heurich M. (Ed.) Ulmer Verlag, Stuttgart, pp. 165–200.
